# Morphological, Biochemical, and Climatological Analysis of Three Moroccan Henna Verities

**DOI:** 10.1155/2019/1418456

**Published:** 2019-05-27

**Authors:** Soukaina El Massoudi, Meryem Benidir, Rachida Chabir, Meryem Benjelloun, Lahsen El Ghadraoui, Faouzi Errachidi

**Affiliations:** ^1^Department of Biology, Laboratory of Functional Ecology and Environment, Faculty of Science and Technology, Sidi Mohammed Ben Abdellah Fes University, P.O. Box 2202, Fes, Morocco; ^2^Laboratory of Pathophysiology and Nutrition and Environment, Faculty of Medicine and Pharmacy, Sidi Mohammed Ben Abdellah University, P.O. Box 1893, Fes, Morocco

## Abstract

In this study, we aimed to evaluate planted Henna in three sites in Morocco, namely, Alnif, Tafraoute Sidi Ali, and Tazzarine. Morphometric study shows that Tafraoute Sidi Ali Henna variety has highest geometric and weight parameters (length of 27.48 mm, width of 10.92 mm, specific mass of 25.1 mg/leaf, leaf area of 51, 53 mm^2^, and rib's number of 9.41) when compared to Alnif and Tazzarine varieties. On the other hand, biochemical analysis shows that Tazzarine Henna variety, in the first rank, is characterized by high levels of total sugars (11.27 g/100 g), reducing sugars (5.59 g/100 g), proteins (4.4 g/100g), lipids (3.05 g/100g), phenolic compounds (31.9 g/100 g), flavonoids (5.68 g/100 g), and tannins (5.5 g/100 g). Chromatographic study shows that Tazzarine Henna variety is rich in monocyclic and polycyclic phenolic compounds. Climatic conditions analysis shows that the morphometric and biochemical diversity is related to hydrous and thermal profiles of studied sites. As a conclusion of this work, we can recommend the use of morphometric analysis and phytochemical and chromatographic analysis to determine the quality of Henna in Morocco and elsewhere.

## 1. Introduction

*Lawsonia inermis* L., commonly known as Henna, belongs to Lythraceae family. This plant, well known for its cosmetic and therapeutic virtues [1], is native to North Africa and South-West Asia [2]. Henna leaves are natural source of colour for hands, feet, fingers, nails, and hair [3]. It contains a pigment called Lawsone (2-hydroxy-1,4-naphthoquinone), mannitol, mucilage, flavonoids (apigenin, luteolin, and quercetin), several phenolic glycosides, coumarins, xanthones, quinoids, beta-cytostrol, lipids, resins, tannins, and catechins. Other compounds derived from the leaves of Henna are derivatives of 5-7 glycosides, gallic acid, acastin, laxanthon 1, and a small amount of alkaloids [4, 5]. Henna biological properties are due mainly to phenolic active ingredients diversity [6]. The latter is as secondary metabolism products drastic climatic conditions adaptation to where this plant is found in a spontaneous way [7]. Qualitative and quantitative analysis investigations may be key tools to verify Moroccan Henna quality. With this in mind, we considered it useful to conduct a study on three varieties of Henna widely used in traditional cosmetics [8]. The lack of Henna normative standard also prompted us to look for simple methods to assess assay potential counterfeits that may be harmful to public health in Morocco [9]. In this context, several intoxications have been reported by the Moroccan poisoning centre caused by counterfeits of Henna [8]. Morocco has established regulation focused on agricultural products distinctive origin and quality [10]. In this optic, we have targeted the characterization of three varieties of Moroccan Henna to align with the said regulations. To do so, we have targeted morphometric, biochemical, and climatological analysis.

## 2. Material and Methods

### 2.1. Plant Material

**Figure 1:**
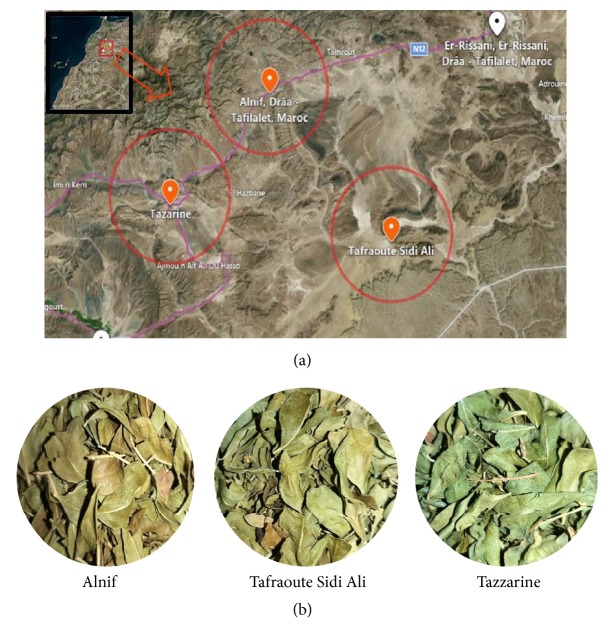
(a) Map situation of the three Moroccan Henna varieties sites used as primary materiel [14]; (b) photographic images of the different varieties.

According to Henna merchant's presurvey we have targeted three samples from Moroccan south-east which are the most commercialized, namely, Alnif Henna, Tafraoute Sidi Ali Henna, and Tazzarine Henna; the geographical situations are illustrated in Figure 1.

### 2.2. Leaves Morphometric Study

Leaves morphometric study [17] has targeted quantitative characteristics, namely, length (L), width (W), Rib's number (RN), leaf specific weight (LSW) and leaf surface (LS) [18]. Geometric characteristics were determined using a digital caliper and an optical magnifier. Leaf specific weight (LSW) was determined from 100 leaves mass according to Wallis [19].

### 2.3. Leaf Analysis

Plant material preparation: dried leaves were milled by electric mill and screened using an airlock (Retsch, standard AFNOR NFX 11504) to obtain a homogeneous powder [20].

Biochemical study: Total sugars were measured by the anthrone method [21] and reducing sugars by the DNS method [22]. Lipids were evaluated by the method described by Khadiga [23]. Protein content was determined by the Lowry method [24]. Phenolic compounds were examined by the Folin-Ciocalteu reagent with some modification [25]. Flavonoids were quantified by the aluminium trichloride method (AlCl_3_) described by Bahroun [26]. Tannins were determined by the method cited by Seigler et al. [27]. All biochemical analyses were done according by dry matter (dm).

Chromatographic study of Henna phenolic compounds extracts: before beginning chromatographic study, we have eliminated lipids by three hexanic extractions. The defatted plant mass was dried at 45°C and has undergone three methanolic extraction [28]. The extract was concentrated under vacuum to get a concentration of 0.5 mg / ml. The three Henna phenolic extracts were chromatographed by using a glass column (50x2.5 cm) containing Sephadex G50 [29, 30] and Lithium chloride buffer (5 mM NaoH, 2.5 mM Licl), as mobile phase. 2 ml fraction was recovered from the column at a flow rate of 1 ml / min. The isolated fractions were analyzed using a UV spectrophotometer at a wavelength of 380 nm [31]. The monocyclic aromatic compounds peak distribution was located by phenol as molecular weight marker, whereas the polycyclic peaks were positioned by quinone.

Climatic study (Emberger bioclimatic Quotient): to inquire about climatic effect on Henna plantations at the three sites (stations) we calculated the Emberger quotient according to the following formulas (Table 1).

Statistical and dimensional analysis: to make a data summary analysis, we used principal component analysis to obtain a multicorrelation between factors by the free software Past 3.2. Variance analysis (ANOVA) of averages was done by MS Excel. All analysis was done in triplicate.

## 3. Results and Discussion

### 3.1. Morphometric Analysis

To get a clearer and more concise idea about the state of the art on Moroccan Henna, we considered that it is useful to collect morphometric parameter to evaluate genetic characterization (Table 2). Henna from Tafraoute Sidi Ali has highest geometric and weight characteristics with length of 27.48 mm, width of 10.92 mm, leaf specific weight of (mg), leaf surface of 51.53 mm^2^, and rib's number of 9.41. Henna from Alnif location has intermediate parameters with a length of 23.41 mm, width of 10.37 mm, leaf specific weight of 15.51 (mg/leaf), leaf surface of 41.1 mm^2^, a rib's number of 8.23. Henna from “Tazzarine” site is characterized by lower values of parameters, namely, length of 21.64 mm, width of 8.99 mm, leaf specific weight of 15.1 mg, and rib's number of 6.09.

Results on leaf dimensions are consistent with those of [32–34] which found similar measurements to our work. Others [35–38] have reported higher values which can reach 50 mm in length and 20 mm in width. Regarding ribs number, our results exceed those found by [34] who found ribs number values between 4 and 5. Remarkable morphometric difference of Moroccan Henna leaves when compared to other countries could be explained by several factors, namely, membership of a bioclimatic stage (semiarid, arid, to pre-Saharan), edaphic factors (shale, limestone,  etc.) [39], stages maturity, sunshine duration, harvest conditions, and other ecological factors [40, 41]. Besides that, this difference could be explained by genetic factors [42].

### 3.2. Biochemical Analysis

Primary and secondary metabolites analysis: biochemical analysis results (Table 3) do not respect the order distinction obtained at the level of the geometric and weight characteristics. Consequently, Tazzarine Henna variety in the first rank and is characterized by high levels of total sugars (11.27 g/100 g), reducing sugars (5.59 g/100 g), proteins (4.4 g/100 g), lipids (3.05 g/100 g), phenolic compounds (31.9 g/100 g), flavonoids (5.68 g/100 g), and tannins (5.5 g/100 g). In second row, Henna Alnif variety has total sugar content of 3.18 g/100 g, reducing sugars of 2.16 g/100 g, proteins of 1.36 g/100 g, lipids of 2.43 g/100 g, phenolic compounds of 25.6 g/100 g, flavonoids of 13.52 g/100 g, and tannins of 3.88 g/100 g. In last row, we find Tafraoute Sidi Ali Henna variety with a total sugar content of 5.5 g/100g, reducing sugars of 2.35 g/100g, proteins of 0.95 g/100 g, lipids of 0.91 g/100 g, phenolic compounds of 27.1 g/100 g, flavonoids of 8.04 g/100 g, and tannins of 4.12 g/100 g. It should be noted that Tazzarine variety is rich in primary and secondary metabolites. Thus, the ecophysiological behaviour does not involve the equilibrium between the two metabolisms (primary and secondary); this makes arise the role of the genetic inheritance in the adaptation to edaphic and climatological conditions.

Primary metabolites analysis shows that total protein and lipids content tested on Henna leaves are lower than those reported by other authors, (10 g/100g (dm). In return total sugars composition, our results are consistent with levels of 10 g/100 g dm cited by [2, 43]. Total phenolic compounds contents in our work agree to those of [43–45] with values ranging from 20 to 32.03 g/100g (dm). The total flavonoids content of the studied Henna agrees with the values of the work [4] and disagrees with those of [43] who cited values of less than 2.5 g/100 g dm, and the work of [44–46] give higher values which reach 47.86 g/100 g dm. Henna leaf powder analysis shows that tannins content agrees with those of [4, 34, 47] around 5 g/100 g dm. Other studies revealed low values (1.79 g/100g dm), [43, 45, 48].

Our results indicate the existence of primary and secondary metabolites variability between the studied samples are depending on several abiotic factors [42, 49]. This variation could be related to the climatic conditions of studied areas [50], leaf maturity, and conditions storage [51]. These hypotheses were verified by consulting climatological data of the three localities. It can be concluded that Henna is strongly characterized by a secondary metabolism closely related to phenolic compounds. The latter seem to have an important role in the characterization of the Henna varieties. This factor has aroused the curiosity to get more information's to link the quality of Henna and the molecular distribution of phenolic compounds.

**Figure 2:**
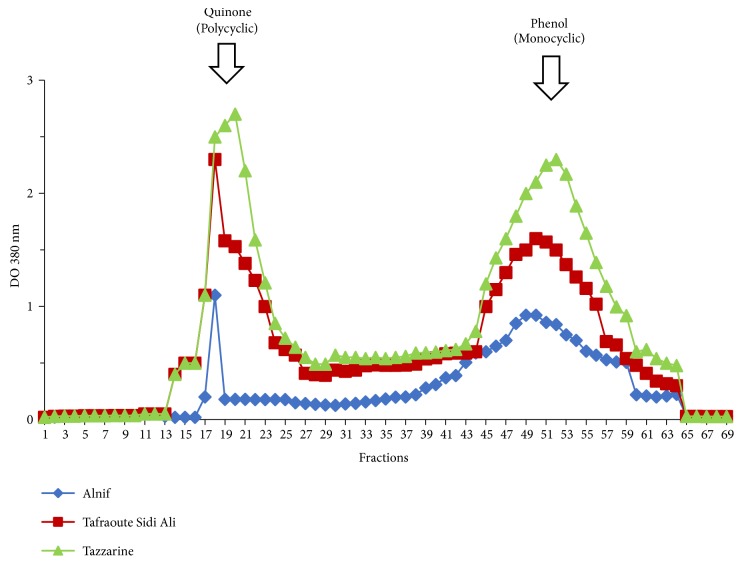
Molecular weight distribution of phenolic compounds of the three Henna varieties studied.

Phenolic compounds molecular weight distribution from the three varieties: preliminary biochemical analysis shows that phenolic compounds are dominant in terms of concentration. This prompted us to conduct more analysis on phenolic compounds in the three Henna studied.  Figure 2 illustrate the molecular weight distribution of the phenolic compounds of the three Henna studied. The first peak (fractions 11 to 27) represents the polymeric phenolic compounds, positioned by quinone molecule used as molecular marker. The second peak (fractions from 35 to 65) represents the monomeric phenolic compounds located by phenol as molecular weight marker. In the present study, we found that Tazzarine Henna represents the richest variety of polycyclic and monocyclic phenolic compounds, followed by Tafraoute Sidi Ali Henna, and finally the Alnif variety. The range of polycyclic molecules of Tazzarine Henna is ten times higher than that of Alnif and almost twice as high as Tafraoute Sidi Ali. We found the same differences in monophenols. Qualitative tests concretized in the colouring tests on skin hands of women volunteers who participated in this evaluation. The dye produced by Tazzarine variety was very striking when compared to Tafraoute Sidi Ali and Alnif verities.

### 3.3. Climatological Analysis

**Figure 3:**
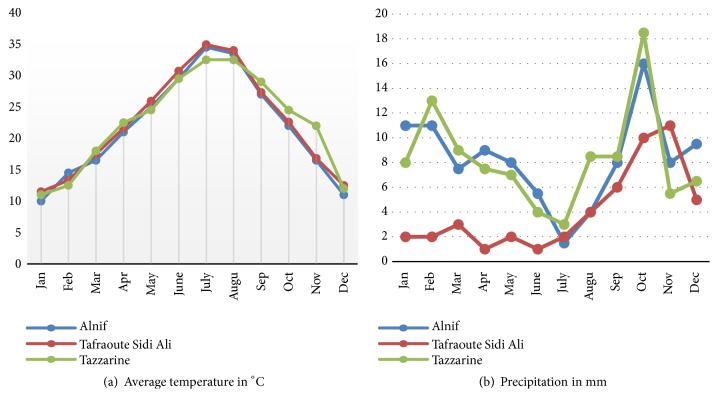
Climatological data (average temperatures and precipitation) of the locations where the three Henna varieties studied are planted [15, 16].

The analysis of mean temperatures and precipitation (Figures 3(a) and 3(b)) shows that the three sites are characterized by different thermal and water profiles. Thus, in order of merit, Tazzarine locality has the highest average temperatures, followed by the Alnif variety and then by the Tafraoute Sidi Ali. Regarding precipitation, Alnif variety is more serviced, relatively, in water when compared to the Tazzarine variety and Tafraoute Sidi Ali variety. These climatological parameters show notable difference found at the three stations that exist in the same Saharan region [52] and that exhibit different microclimatic specifications that give elements of answers on the biochemical diversity caused by equilibria between a primary and secondary metabolism. The plant studied is considered as an important source of phenolic compounds, carbohydrates, and proteins [37] and can serve as a natural source of antioxidants, giving curative property against gastric diseases, venereal diseases, dermatoses, and high blood pressure [53, 54].

Emberger quotient analysis done by three methods shows that the formulas proposed by [12, 13] give similar results compared to that described by [11]. Among the bioclimatic indices traditionally used in North Africa and elsewhere in the Mediterranean area [55–57], there is the Emberger bioclimatic index (Table 4). This considers the annual precipitation (P), the mean maxima temperature (Tm) of the hottest month (M in °C or °K), and the average minimum temperature of the coldest month (m in °C or °K) [11].

The corrected quotients Q2′ and Q2′′ proposed by [12, 13], particularly adapted to the Mediterranean's regions, reflect the relationship between the amount of precipitation and the average of thermal extremes (Hottest and coldest months), all corrected by the extreme thermal amplitude (M - m). Emberger noted that thermal amplitude is an important factor in the distribution of vegetation. The combination of the pluviothermal quotients obtained (Q2′′) and the minimum temperatures of the coldest month (m) (limiting factor) with the observations made on the distribution of Mediterranean vegetation and more particularly in Morocco led Emberger to subdivide the climacteric area in characteristic zones by growing aridities from top to bottom. These are the “bioclimatic stages of vegetation” selected as follows: Saharan, arid, semiarid, subhumid, wet, and perwet. Our results show that Moroccan Henna belongs to the Saharan bioclimatic stage according to the Emberger quotient [11] and to the extreme arid climate for Alnif and Tazzarine and Saharan for Tafraoute Sidi Ali according to Emberger modified by [12, 13].

**Figure 4:**
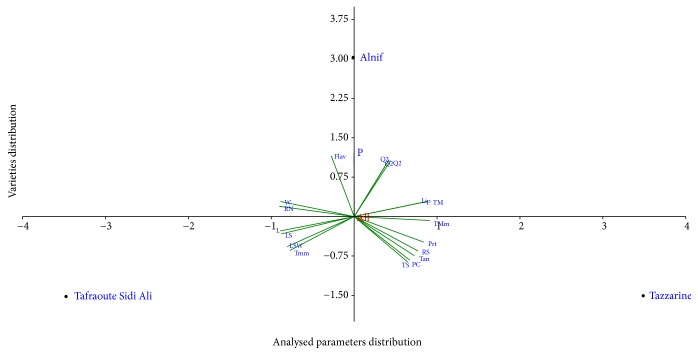
Dimensional analysis of the morphometric, biochemical, and climatological data of the three varieties of Henna. L: length, W: width, LSW: leaf specific weight, LS: leaf surface, RN: rib's number, TS: total sugars, RS: reducing sugars, Prt: proteins, Lip: lipids, PC: phenolic compounds, Flav: flavonoids, Tan: tannins, TM: mean of temperature, Tmm: mean of minimal temperature, TMm: mean of maximal temperature, P: precipitation; Q2: Emberger quotient 1; Q2′: Emberger quotient 2, Q2′′: Emberger quotient 2′.

The results of the principal component analysis summarized in the graph shown in Figure 4 have shown that the spatial distribution is related to Henna composition. Thus, lipids, total sugars, reducing sugars, proteins, phenolic compounds, and tannins are positively correlated with axis 1 with Tazzarine Henna, which means that this variety is characterized by biochemical parameters; however length, width, leaf specific weight, leaf surface, and rib's number are negatively correlated with axis 1 and is consistent with the Tafraoute Sidi Ali Henna, which means that Tafraoute Sidi Ali is more characterized by morphometric parameters; in other the climatological parameters and the flavonoid content are consistent with Alnif Henna positively contributing to axis 2, which means that the Alnif variety is influenced by climatological parameters.

These distributions show that the biochemical, morphological, and climatological analysis carried out makes it possible to group the varieties of Henna studied and to characterize them according to their biochemical content and their morphologies. From this it can be concluded that Henna is strongly characterized by a secondary metabolism closely related to phenolic compounds. The latter seem to have an important role in the characterization of the Henna varieties studied. This factor has aroused in us the curiosity to have more information to link the quality of Henna and the molecular distribution of phenolic compounds.

## 4. Conclusion

Morphometric study shows that Tafraoute Sidi Ali variety presents the highest geometric and weight parameters followed by Alnif and Tazzarine varieties. On the other hand, the biochemical study shows that the Tazzarine variety is characterized by higher phenolic compound contents, and the variety of Alnif is characterized by higher value of flavonoids. This study shows that the Tazzarine variety is characterized by the highest total and reducing sugars content. Chromatographic study shows that the Tazzarine variety is the richest in monocyclic and polycyclic phenolic compounds. As a conclusion of this work, we can recommend the use of morphometric analysis and biochemical and chromatographic analysis to determine the quality and distinctive signs of origin according to the regulation in force of Henna in Morocco. In perspective, we aim to begin phenolic compounds identification to determine the fractionation of Lawsone in all phenolic compounds as quantitative coefficient of the quality of Henna we are now targeting Lawsone as means of Henna enhancement in Morocco.

## Figures and Tables

**Table 1:** Emberger's bioclimatic Quotient formulas.

Formulas	References	Climatological parameters
Q2 = 200*∗*P/(M2-m2) Q2′= 3.43P*∗*/(M-m)	Emberger [11] Stewart [12]	P: Annual rainfall in mm/m2/year M: Maximum temperature of the hottest month in °K m: Minimum temperature of the coldest month in °K
Q2′′= 2000P/(M+m+546.4) *∗* (M-m)	Mokhtari et al. [13]	P: Annual rainfall in mm/m2/year M: Maximum temperature of the hottest month in °C m: Minimum temperature of the coldest month in °C

**Table 2:** Morphometric analysis studied of Henna varieties.

Parameters	Alnif	Tafraoute Sidi Ali	Tazzarine
Length (L) (mm)	23.41	27.48	21.64

Width (W) (mm)	10.37	10.92	9

Leaf Specific Weight (LSW) (mg)	15.51	25.1	15.1

Leaf Surface (LS) (mm^2^)	42.1	51.53	38.82

Rib's number (RN)	8.23	9.41	6.1

**Table 3:** Biochemical analysis of the Three Henna varieties studied.

parameters (g/100g dm)	Alnif	Tafraoute Sidi Ali	Tazzarine
Total sugars (TS)	3.18	5.4	11.27

Reducing sugars (RS)	2.16	2.35	5.59

Proteins (Prt)	1.36	0.95	4.4

Lipids (Lip)	2.43	0.91	3.05

Phenolic compounds (PC)	25.6	27.1	31.9

Flavonoids (Flav)	13.52	8.09	5.68

Tannins (Tan)	3.88	4.12	5.5

**Table 4:** Climatological analysis of the three Henna varieties studied.

	Parameters	Alnif	Tafraoute Sidi Ali	Tazzarine
Climatological parameters	Mean of temperature TM (°C)	20.11	14.39	22.43
	Mean of minimal temperature (Tmm) (°C)	13.75	30.53	14.39
	Mean of maximal temperature (TMm) (°C)	26.00	22.38	30.53
	Precipitation (P) (mm)	13.08	4.08	4.08

Emberger quotient	Q2 [11]	9.09	2.62	5.60
	Q2′ [12]	18.49	5.25	11.63
	Q2′′ [13]	17.64	5.08	10.87

## Data Availability

No data were used to support this study.
